# Spatial frequency domain Mueller matrix imaging

**DOI:** 10.1117/1.JBO.27.12.126003

**Published:** 2022-12-14

**Authors:** Joseph Chue-Sang, Maritoni Litorja, Aaron M. Goldfain, Thomas A. Germer

**Affiliations:** a University of Maryland, Department of Chemistry and Biochemistry, College Park, Maryland, United States; b National Institute of Standards of Technology, Sensor Science Division, Gaithersburg, Maryland, United States

**Keywords:** brain tissue, Mueller matrix, polarimetry, scattering, spatial frequency domain imaging, tissue anisotropy

## Abstract

**Significance:**

Mueller matrix polarimetry (MMP) and spatial frequency domain imaging (SFDI) are wide-field optical imaging modalities that differentiate tissue primarily by structure alignment and photon transport coefficient, respectively. Because these effects can be related, combining MMP and SFDI may enhance tissue differentiation beyond the capability of each modality alone.

**Aim:**

An instrument was developed to combine MMP and SFDI with the goal of testing whether it enhances contrast of features in reflection mode.

**Approach:**

The instrument was constructed using liquid crystal elements for polarization control, a digital light processing projector for generating sinusoidal illumination patterns, and a digital camera for imaging. A theoretical analysis shows that the SFD Mueller matrix is complex-valued and does not follow the same behavior as a regular Mueller matrix. Images were acquired from an anisotropic tissue phantom, an optical fiber bundle, and cerebellum, thalamus, and cerebrum tissues.

**Results:**

The measurement results suggest that singly scattered, few scattered, and diffusely scattered photon paths can be distinguished in some of the samples investigated. The combined imaging modality yields additional spatial frequency phase information, which highlights paths having only a few scattering events.

**Conclusions:**

The combination of MMP and SFDI offers contrast mechanisms inaccessible by each modality used alone.

## 1 Introduction

The Mueller matrix characterizes the polarization-sensitive reflection or transmission properties of a material for a given optical path.^1^^,^^2^ From the Mueller matrix, specific polarimetric properties, such as retardance, diattenuation, and depolarization, can be determined. Highly scattering materials, such as biological tissues and optical phantoms intended to mimic them, generally exhibit a strong depolarizing response.^3^ Retardance and diattenuation can be observed in anisotropic media, such as connective tissue, muscle, and brain tissue.^4^^–^^9^ Mueller matrix measurements in transmission can be interpreted in terms of a differential formalism yielding birefringence or diattenuation.^6^^,^^10^^–^^12^ Coherent Stokes vector and Mueller matrix methods in transmission have shown differentiation between structures in different biological tissues.^13^^–^^18^ Changes in retardance and diattenuation can reflect disruptions in connective tissue, and Mueller matrix polarimetry (MMP) has been shown to be useful for detecting changes in collagen alignment, such as in cervixes during pregnancy or cancer.^4^^,^^5^^,^^19^ The MMP is commonly conducted using full field illumination, allowing it to characterize a large sample region. The measured Mueller matrix results from all the paths the light takes, including those that have penetrated deeply into the sample. Biological tissues, however, are rarely uniform in their optical properties throughout a volume,^20^ and discrimination between contributions from surface and deep tissues can be important. Organization of structures is important in distinguishing healthy and diseased tissue, and MMP is particularly sensitive to changes in structural organization.^19^ One issue with MMP is that deeply penetrating photons can impede detection of those photons that have only interacted with shallow tissues. Confocal MMP imaging has been one technique that has been attempted to circumvent this issue.^21^^,^^22^

Spatial frequency domain imaging (SFDI) has the potential for controlling the penetration depth of investigating photons.^23^ In SFDI, an incoherent sinusoidal spatially periodic pattern is projected onto the material, and the amplitude of the reflected modulation is recorded as the phase of that pattern is varied. In this manner, only photons whose path lengths are shorter than the period of the pattern are sensed, making depth sensitivity possible. A high spatial frequency can mitigate the amount of noise in an image by reducing the multiply scattered photons detected in favor of singly scattered photons and providing better image contrast.^23^^,^^24^ Like MMP, SFDI provides full field imaging, allowing for a macroscopic sample area to be investigated. Measurements of the polarization of light scattered a distance from a point of illumination have been performed in the past, but the method is less amenable to full field imaging.^25^ Because SFDI is typically operated in reflection mode, it can be employed noninvasively.

In this paper, we describe the combination of SFDI and MMP imaging and present some example measurements. Whereas polarized SFDI has been studied in the past,^24^^,^^26^ we expand on this idea by measuring full Mueller matrices in the SFD. In terms of the instrumentation, combining SFDI with MMP is relatively straightforward, as they share a number of components, and each modality only needs the addition of those specific to the other. Both modalities are also inexpensive compared with some other imaging modalities, such as confocal imaging or optical coherence tomography. By blending these methods, we combine the structural sensitivity of MMP with the photon path length selectivity of SFDI.

This work expands on two previous studies,^27^^,^^28^ which first explored this combination of modalities, by improving upon the data acquisition and analysis and expanding the range of materials studied. For example, a number of details need to be addressed to ensure that unwanted artifacts do not appear. In addition, it is found that a SFD Mueller matrix differs from a regular Mueller matrix, in that it does not follow the same rules and is complex-valued.

In Sec. 2, we develop the theory associated with the combined SFDI and MMP modalities. In Sec. 3, we describe the instrumentation for our measurements. Our data reduction methods are then outlined in Sec. 4. The samples we use to demonstrate the measurements are described in Sec. 5. In Sec. 6, we show the results of the measurements. We discuss the combined method in Sec. 7 and make some conclusions in Sec. 8.

## 2 Theory

The SFDI is often interpreted using an approach based upon the diffusion equation.^23^ However, this approach does not account for singly scattered radiation and is not valid when the spatial frequency is on order of or greater than the transport coefficient. Polarization imaging often is particularly sensitive to the contributions of singly scattered radiation, and as a result, we find that a different approach is needed. In the theory of spectrophotometry,^29^^,^^30^ the bidirectional scattering-surface reflectance distribution function (BSSRDF) S expresses the relationship between the radiance $dL_{r}$ reflected from a surface at a location x,y and propagating in a direction θ^, when radiant power ${d\Phi }_{i}=E_{i}{\text{d}x}^{\prime }{\text{d}y}^{\prime }$ ($E_{i}$ is the incident irradiance) is incident from a direction θ^′ onto the surface at a location $x^{\prime },y^{\prime }$. When considering polarization, the radiation properties are naturally represented as 4-element Stokes vectors, and the BSSRDF as a 4×4 Mueller matrix. Thus, we make the transformation to Stokes vector quantities, ${\text{d}L}_{\text{r}}\to {\text{d}L}_{\text{r}}$, ${d\Phi }_{\text{i}}\to {d\Phi }_{\text{i}}$, and $E_{i}\to E_{\text{i}}$, the Mueller matrix S→S, and express this relationship as

$$\text{d}L_{\text{r}}=S\text{d}\Phi_{\text{i}}=SE_{\text{i}}\text{d}x^{\prime }\text{d}y^{\prime }.$$ (1)

In Eq. (1), we abbreviate $dL_{\text{r}}={\text{d}L}_{\text{r}}(x,y)$, ${d\Phi }_{\text{i}}={d\Phi }_{\text{i}}(x^{\prime },y^{\prime })$, $E_{\text{i}}=E_{\text{i}}(x^{\prime },y^{\prime })$, and $S=S(x,y;x^{\prime },y^{\prime })$. We also drop the directional coordinates θ^ and θ^′, as well as the wavelength dependency, as they are generally fixed in our measurements. Whereas the phenomenological BSSRDF does not describe the physics of the scattering process, it successfully describes the radiation redistribution caused by the material under conditions of incoherent illumination. It is also straightforward to model using Monte Carlo methods.^31^^–^^33^ It should be borne in mind that regular Stokes vectors obey the inequality, for example

$$L_{\text{r},1}\geq {\left(L_{\text{r},2}^{2}+L_{\text{r},3}^{2}+L_{\text{r},4}^{2}\right)}^{1/2},$$ (2)

where $L_{\text{r},j}$ is the j’th element of $L_{\text{r}}$. Stokes vectors that obey Eq. (2) are either fully polarized (in the case of equality) or the sum of two (in the case of inequality) fully polarized Stokes vectors. The Mueller matrix S obeys the requirement that it must map the space of valid Stokes vector radiant powers onto the space of valid Stokes vector radiances. In addition, S must satisfy the Cloude requirement that it be the convex sum of up to four nondepolarizing Jones–Mueller matrices.^34^^,^^35^

In SFD Mueller matrix imaging, we illuminate the surface with an incoherent Stokes vector irradiance

$$E_{\text{i}}(x^{\prime },y^{\prime })=E_{0}[1+\cos(2\pi fx^{\prime }+\phi )],$$ (3)

where f is the spatial frequency, ϕ is a phase, and $E_{0}$ is a Stokes vector irradiance. Note that we could generalize f to include the direction along which illumination is oriented, and in some of our measurements, we modulated the irradiance along $y^{\prime }$. The radiance emitted by the surface is determined by integrating Eq. (1)

$$L_{\text{r}}=\iint SE_{\text{i}}dx^{\prime }dy^{\prime }.$$ (4)

If we insert the irradiance Eq. (3) into Eq. (4), and assume that the material is uniform [so that $S(x,y;x^{\prime },y^{\prime })=S(x-x^{\prime },y-y^{\prime })$], the radiance is given as

$$L_{\text{r}}=f_{\text{r}}E_{0}+\int S_{1}(x-x^{\prime })E_{0}\;\cos(2\pi fx^{\prime }+\phi )dx^{\prime },$$ (5)

where the line spread function is given as

$$S_{1}(x-x^{\prime })=\int S(x-x^{\prime },y-y^{\prime })dy^{\prime },$$ (6)

and $f_{r}$ is the Mueller matrix BRDF.

In SFDI, we vary the phase ϕ of the irradiance and measure the modulation of the radiance. To demodulate the radiance, we evaluate

$${\overset{˜}{L}}_{\text{r}}(f)=\frac{1}{\pi }\int_{0}^{2\pi }L_{\text{r}}\;\exp(2\pi \text{i}fx+i\phi )d\phi .$$ (7)

If we insert Eq. (5) into Eq. (7), and perform the integral, we obtain

$${\overset{˜}{L}}_{r}(f)=\int S_{1}(x-x^{\prime })E_{0}\;\exp[2\pi if(x-x^{\prime })]dx^{\prime }.$$ (8)

The SFD Stokes radiance ${\overset{˜}{L}}_{\text{r}}(f)$ does not obey the same inequality Eq. (2) as the regular Stokes vector $L_{r}$, because it is not a convex sum of regular Stokes vectors. Furthermore, it can be complex-valued. Negative radiance can be interpreted as radiance emitted from locations in the troughs of the irradiance, whereas complex parameters result from the polarization of radiation emitted to one side being different from that emitted from the other. At f=0, the integral in Eq. (8) evaluates to

$${\overset{˜}{L}}_{r}(0)=f_{r}E_{0}.$$ (9)

We thus define the SFD BRDF

$$F_{r}(f)=\int S_{1}(x-x^{\prime })\exp[2\pi \text{i}f(x-x^{\prime })]dx^{\prime },$$ (10)

so that

$${\overset{˜}{L}}_{\text{r}}(f)=F_{\text{r}}(f)E_{0}.$$ (11)

We notice a couple interesting properties of the matrix $F_{r}(f)$. Because the quantity is a Fourier transform of a regular Mueller matrix, it is not necessarily physically realizable as one. That is, Mueller matrices can only be safely added with positive coefficients. Matrices that are physically unrealizable have the property that they can overpolarize or yield negative power. If radiation is incident at one location and exits from another location, the SFD Stokes radiance and the SFD BRDF may not follow the customary rules that apply to regular Stokes vectors or Mueller matrices. We use the term SFD Mueller matrix, or SFD BRDF to represent the generalization of the Mueller matrix and emphasize that its properties at nonzero spatial frequency differ from those of regular Mueller matrices.

A regular Mueller matrix can be expressed^34^^,^^35^ as a covariance matrix H, whose ordered eigenvalues obey $\lambda_{0}\geq \lambda_{1}\geq \lambda_{2}\geq \lambda_{3}\geq 0$. The polarimetric purity indices,^36^

$$P_{1}=\frac{\lambda_{0}-\lambda_{1}}{\sum_{i}\lambda_{i}},\;P_{2}=\frac{\lambda_{0}+\lambda_{1}-2\lambda_{2}}{\sum_{i}\lambda_{i}},\;P_{3}=\frac{\lambda_{0}+\lambda_{1}+\lambda_{2}-3\lambda_{3}}{\sum_{i}\lambda_{i}},$$ (12)

obey the inequality

$$0\leq P_{1}\leq P_{2}\leq P_{3}\leq 1.$$ (13)

In the SFD, some of the eigenvalues $\lambda_{i}$ can be negative or even complex-valued, and the polarimetric purity indices no longer obey the inequality in Eq. (13). In particular, $P_{3}$ can be greater than unity.

If the BSSRDF is asymmetric, i.e., $S_{1}(x-x^{\prime })\neq S_{1}(x^{\prime }-x)$, then the matrix $F_{\text{r}}(f)$ will be complex-valued. Note that the absolute phase of the signal is not necessarily known in an experiment. For example, for nonnormal illumination and viewing, the surface topography and the depth of the scatterer can affect the overall phase of the image compared with the illumination. As a result, we correct the phase by requiring that the upper-left element ($M_{11}$) be real-valued.

## 3 Instrumentation

**Fig. 1:**
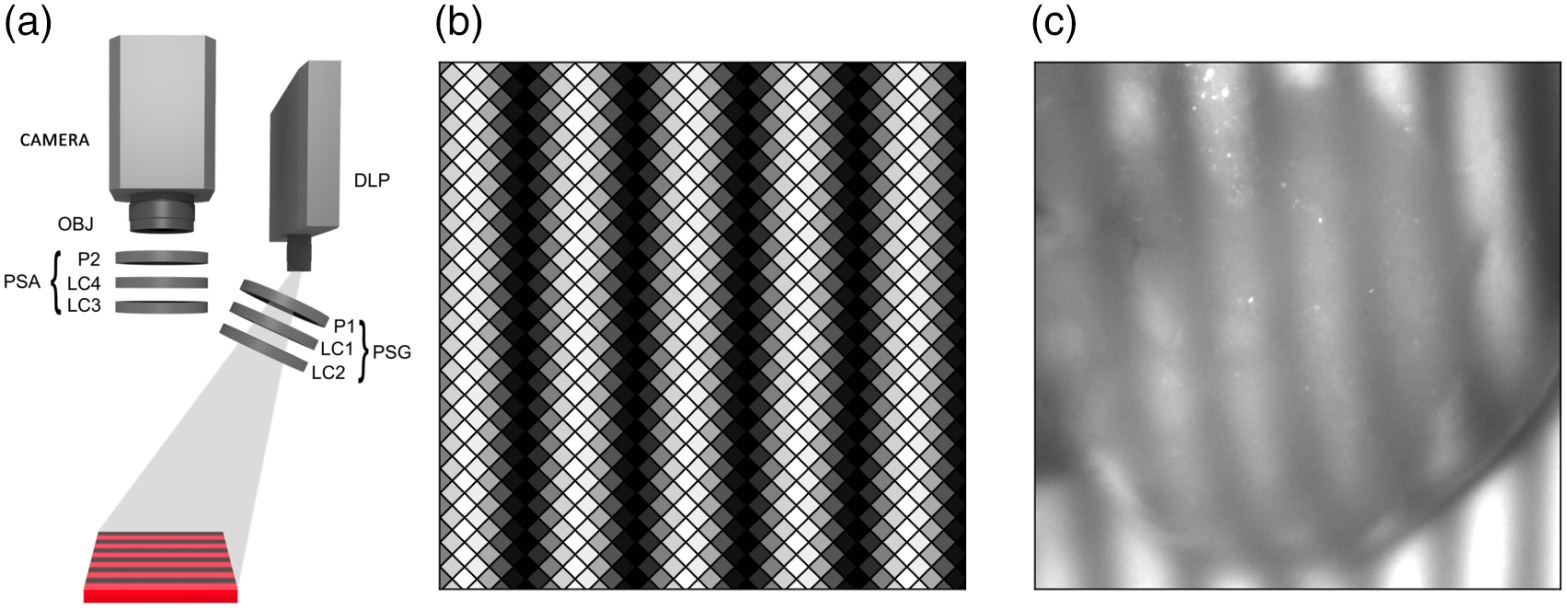
(a) SFD Mueller matrix imaging system: PSG with polarizer (P1) and two LC retarders (LC1 and LC2), DLP projector, PSA with polarizer (P2) and two LC retarders (LC3 and LC4), objective (OBJ), and sCMOS camera (CAMERA). (b) A representation of how the sinusoid pattern for $f=5\;{\mathrm{cm}}^{-1}$ is displayed by the DLP projector pixels. (c) One of the 96 images acquired of bovine thalamus for vertical sinusoidal illumination at $f=5\;{\mathrm{cm}}^{-1}$.

Figure 1(a) shows an illustration of the SFD Mueller matrix imaging system. Structured illumination is generated using a digital light processing (DLP) projector (Texas Instruments, model Lightcrafter 4500), which uses three light emitting diodes (LEDs) with wavelengths centered at 455, 520, and 630 nm as well as digital micromirror devices to pulse-width modulate the light source according to the image and settings sent to the device. All images in this study were taken using only the 630-nm LED, because using a single wavelength simplifies the operation of the instrument. This LED’s spectrum was measured to have a full width at half maximum (FWHM) of 16 nm centered at 628 nm. We used a 10-nm bandpass filter centered at 633 nm to narrow the spectral width. The spectrum measured after applying the filter had a FWHM of 6.5 nm centered at 632 nm. The illumination is ∼20  deg from the sample surface normal. The DLP has 1140 pixels in the vertical direction and 912 pixels in the horizontal direction arranged in a diamond pattern [see Fig. 1(b)] with the rows being separated by half the distance of the columns and offset by half a pixel horizontally. To ensure that a horizontal or vertical sinusoidal projected pattern is commensurate with the array, to have a common number of phases, and to have a smoothly modulating pattern, spatial frequencies were restricted to those having periods with multiples of three pixels and N=6 phases were used, twice that necessary,^37^ providing some oversampling and improving complex-valued demodulation. The periods ∞, 24, 18, 12, 9, and 6 pixels on the projector correspond to $f=(0,1.2,1.7,2.5,3.3,5.0)\;{\mathrm{cm}}^{-1}$ at the sample plane. Because three pixels are not truly commensurate with the array, demodulation was often poor and results from that spatial frequency are not presented. The projected sinusoidal pattern can be displayed in either the horizontal or vertical direction, both of which are used in this manuscript. An example image can be seen in Fig. 1(c), where a vertical sinusoidal pattern is used to illuminate a section of bovine thalamus at $f=5\;{\mathrm{cm}}^{-1}$. The total area illuminated by the projector at the sample plane (>17  cm diameter) is much larger than the imaged area (1.1  cm×1.1  cm) and illuminates surrounding objects and the support structure. To avoid the effects of stray light, especially for f=0 illumination, the projected pattern was cropped in software to illuminate only the area close to the sample.

The sample is imaged normal to the sample with a 50-mm focal length objective (Edmund Optics, model 59-873) onto a monochrome 16-bit 2048×2048 scientific complementary metal oxide semiconductor (sCMOS) camera with $(6.5\times 6.5)\;{\mu m}^{2}$ pixel area (PCO, model pco.panda 4.2). The system has a full field of view at the sample plane of 7.5  cm×7.5  cm, though all images presented here are cropped to 1.1  cm×1.1  cm (300  pixels×300  pixels). The camera is synchronized to the projector and its exposure time is set as a multiple number of milliseconds. Thus, to avoid interference of the camera exposure time with the pulse width modulation of the projector (frame rate $120\;s^{-1}$), the camera exposure time was chosen to be a multiple of 25 ms (3  ms×8.33  ms).

The polarization state generator (PSG) and the polarization state analyzer (PSA) each consist of a polarizer and a pair of nematic liquid crystal (LC) retarders (Meadowlark Optics). The axes of the LCs are aligned nominally at 27.4 deg and 72.4 deg with respect to the polarizer, which is the optimum configuration.^38^ Each of the four LCs were switched between two retardance states (determined by applied voltage) providing the 16 different combinations needed to measure a full Mueller matrix. The polarimeter was calibrated with the eigenvalue method described by Compain et al. using three samples:^39^ air (no sample, in transmittance), a Glan–Thompson polarizer (in transmittance), and a Si wafer with an ∼1000-nm-thick ${\mathrm{SiO}}_{2}$ layer acting as a mirror with retardance and diattenuation (in reflection with an incident angle of 60 deg). This eigenvalue method yields a calibration that is valid for both transmission and reflection measurement geometries. The 10-nm spectral bandwidth filter, mentioned earlier, was necessary to achieve an acceptable calibration due to the native bandwidth of the light source producing a reduction matrix with high dispersion. The standard deviation of all the reference measurements across all elements of the normalized Mueller matrix was 0.014. We believe we can use this standard deviation as the standard uncertainty in subsequent measurements. Control of the projector, the LCs, and the camera was performed using a program written in MATLAB (MathWorks) software.

The final array of 672 images (7 spatial frequencies × 6 phases × 16 PSG/PSA combinations) took 4.2 min to acquire, if only imaged with one spatial frequency orientation. This time was significantly improved from the previous iteration of the instrument, mostly due to the use of a faster camera and synchronization of it with the projector.^27^ The response and settling time of the LC retarders is the predominant factor limiting acquisition time.

Figure 1(c) shows one acquired image, having a specific phase and PSG/PSA combination, for one of the samples (bovine thalamus, described later) for vertical sinusoidal illumination at $f=5\;{\mathrm{cm}}^{-1}$. Most of the phase variation observed in Fig. 1(c) is a result of the topography and shape of the specimen. The lack of sharp optical contrast is a result of diffuse scatter in the medium.

## 4 Data Reduction

In this work, we perform measurements of the radiance (an image) $L_{r,jk}$ for a set of 16 combinations of PSG and analyzer configurations (denoted by index) for each of six illumination phases (denoted by index j). We begin by demodulating the signal by substituting the integral in Eq. (7) with a sum, ignoring the overall phase factor exp(2πifx)

L^r,k(f)=1N∑j=1NLr,jk(f) exp(i2πjN), (14)

where N is the number of phases. Note that this demodulation scheme preserves phase, unlike that typically used for SFDI, which only measures amplitude.^23^ We then apply a data reduction matrix $R_{k}$ to the L^r,k(f) to yield the complex SFD Mueller matrix

M(f)=∑k=116RkL^r,k(f). (15)

The data reduction matrix is determined from the eigenvalue calibration procedure.^39^ The SFD Mueller matrix carries with it the phase factor exp(2πifx), in addition to phase that results from topography and varying depth. We thus remove the overall phase by forcing $M_{11}$ to be real-valued by applying the transformation

$$M(f)\leftarrow M(f)\frac{\left|M_{11}(f)\right|}{M_{11}(f)}.$$ (16)

Finally, the SFD Mueller matrix BRDF ${\overset{˜}{f}}_{r}(f)$ can be related to the measured matrix^27^ M, as follows:

$$F_{\text{r}}(f)=M(f)\times \frac{f_{\text{r}}^{\text{d}}}{\pi M_{11}^{\text{d}}(0)}\times \frac{M_{11}^{\text{r}}(0)}{M_{11}^{\text{r}}(f)},$$ (17)

where the first correction factor uses a measurement $M^{d}(0)$ of a diffuse reflector of known BRDF $f_{r}^{d}$ in the same incident and viewing geometry to provide a scale in inverse steradians, and the second correction factor uses a measurement of $M^{r}$ of a nondiffusive surface, such as a rough metal, to account for the modulation transfer function of the instrument.

Further analysis of the SFD Mueller matrix is complicated by the fact that the matrix is not a convex sum of Jones–Mueller matrices and that it can be complex-valued. In those cases where the imaginary part of the matrix is negligible and the matrix is realizable, we can apply the Lu–Chipman decomposition,^40^ expressing the matrix as the ordered product of a diattenuator $M_{\text{D}}$, a phase retarder $M_{\text{R}}$, and a depolarizer ${\text{M}}_{\Delta }$

$$M=M_{\Delta }M_{R}M_{D}.$$ (18)

From these individual elements, we can extract the linear diattenuation, diattenuation orientation, linear retardance, retardance orientation, and depolarization.^1^

The diattenuation D shown in this paper is on a scale from 0 to 1, where D=0 indicates that the material has no selectivity of the orientation of the polarization plane of linearly polarized light, whereas D=1 indicates one polarization state is entirely attenuated. Anisotropic materials often display some degree of diattenuation. In addition, the 20 deg incident angle geometry can impose some effective diattenuation.

## 5 Samples

We performed measurements on five samples. The first sample was a phantom with an anisotropic scatterer embedded at a graded depth in an isotropic scattering medium. The phantom fabrication procedure is detailed elsewhere,^41^ but in brief, polydimethylsiloxane (PDMS) (Corning, model Sylgard 184 Elastomer) was used as a base to create the scattering and absorbing media. Titanium dioxide (Atlantic Equipment Engineers, TI-602, size 0.3 to 1.0  μm) and carbon black particles (Atlantic Equipment Engineers, FE-603, size 1 to 2  μm) were mixed within the PDMS to act as scatterers and absorbers, respectively. The scatterer and absorber were kept in stock suspensions of 1% mass ratio ${\mathrm{TiO}}_{2}$ powder in pure PDMS and 0.1% mass ratio carbon black powder in pure PDMS, respectively. The absorption coefficient ($0.4{\;\mathrm{cm}}^{-1}$) and the reduced scattering coefficient ($7.5{\;\mathrm{cm}}^{-1}$) were determined from diffuse reflectance and transmittance measurements, together with an inverse adding-doubling algorithm.^42^ Para-aramid fibers obtained from cladding of furcation cables (Thorlabs, FT030) were used as anisotropic scatterers. The fibers were fixed to a glass microscope slide with adhesive. The slide was then inclined in a Petri dish acting as a mold and filled with the PDMS solution. The para-aramid fibers were set so that one end of the fibers was exposed while the other end was submerged. In this way, the thickness of the scattering/absorbing PDMS media above the fibers gradually increases from 0 to 4 mm from one end of the slide to the other. Only a portion of the slide was imaged, so the results we present had fibers embedded less than about 1 mm. A glossy white ceramic tile was used as the background when this sample was imaged. The use of the tile was chosen to demonstrate the reduced impact of a highly depolarizing background.

Our second sample was an optic fiber bundle (image conduit). The fiber bundle had a diameter of 3.2 mm, contained 50,419 individual fibers having 12-μm cores, and was approximately 25 mm long (Edmund Optics, model 53-839). The individual fibers are expected to act as anisotropic scatterers and scatter primarily perpendicular to the fiber axes. The fibers may also exhibit some form birefringence or diattenuation. Furthermore, light entering the bundle may internally reflect and be reemitted from a different location. We imaged the fiber bundle resting on its side upon the glossy white ceramic tile.

The remainder of our samples were mammalian brain tissues: a caprine (goat) cerebellum, a bovine (cow) thalamus, and a bovine cerebrum. The brains were acquired intact from a local Halal abattoir. Sections of brain were then excised and placed in a Petri dish for imaging within an hour of acquiring the brains. The samples were all greater than 1.5 cm thick.

## 6 Results

### 6.1 Para-Aramid Fiber Embedded in PDMS

**Fig. 2:**
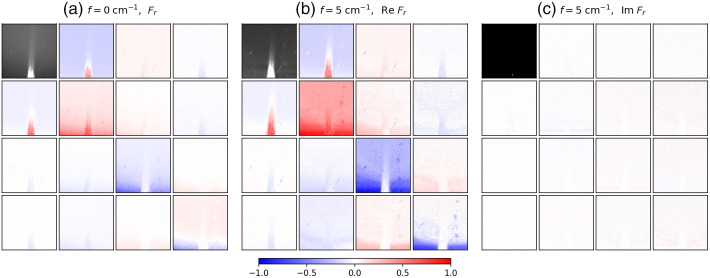
SFD Mueller matrix BRDFs from the para-aramid fiber embedded in a PDMS-based scattering and absorbing phantom: (a) the Mueller matrix BRDF at $f=0\;{\mathrm{cm}}^{-1}$, (b) the real part of the SFD Mueller matrix BRDF at $f=5\;{\mathrm{cm}}^{-1}$, and (c) the imaginary part of the SFD Mueller matrix BRDF at $f=5\;{\mathrm{cm}}^{-1}$. The maximum value of the black and white scale used for $F_{\text{r},11}$ at $f=0\;{\mathrm{cm}}^{-1}$ is twice the maximum value for $F_{\text{r},11}$ of $f=5\;{\mathrm{cm}}^{-1}$. The scale for the normalized SFD Mueller matrix elements is shown at the bottom of the figure. The spatial frequency sinusoidal pattern for $f=5\;{\mathrm{cm}}^{-1}$ was oriented horizontally. The field of view of the images is 1.1  cm×1.1  cm.

Figure 2 shows a subset of the SFD Mueller matrix images for the para-aramid fiber embedded in the PDMS phantom (full datasets are available online, see Data Availability below). Figure 2(a) shows the Mueller matrix BRDF at $f=0\;{\mathrm{cm}}^{-1}$, whereas Figs. 2(b) and 2(c) show the real and imaginary parts, respectively, of the SFD Mueller matrix BRDF illuminated with a horizontal sinusoidal pattern at $f=5\;{\mathrm{cm}}^{-1}$. All but the $M_{11}$ terms are shown normalized to their respective $M_{11}$, which are forced to be real-valued. As $M_{11}$ is defined to be real, it is zero in the imaginary part of the SFD Mueller matrix [see Fig. 2(c)]. The thickness of the PDMS is very thin at the bottom of the images and increases to about 1 mm at the top of the image. The para-aramid fibers, which are embedded within the PDMS phantom, stretch vertically through the center of the image and are most visible at the bottom, where the PDMS is thinnest and some of the fibers are exposed at the edge. The $M_{11}$ at $f=5\;{\mathrm{cm}}^{-1}$ has lower intensity than that at $f=0\;{\mathrm{cm}}^{-1}$, which is typical for SFDI and is due to the filtering of diffusing photons. The imaginary part of the $f=5\;{\mathrm{cm}}^{-1}$ SFD Mueller matrix is negligible for this sample, suggesting that the scattering is primarily symmetric. The real part of the $f=5\;{\mathrm{cm}}^{-1}$ SFD Mueller matrix has somewhat higher contrast than its $f=0\;{\mathrm{cm}}^{-1}$ counterpart. Most noticeably, the vertical extent to which the fibers can be observed in the SFD Mueller matrix is increased at the higher spatial frequency. The spatial frequency imaging filters the diffusing photons, which are more depolarizing, yielding the increased image contrast in the SFD Mueller matrix elements. Because the scatter from the fibers is more dependent upon polarization than the scattering from the PDMS, the single scattering from the fibers can be observed from deeper in the material.

**Fig. 3:**
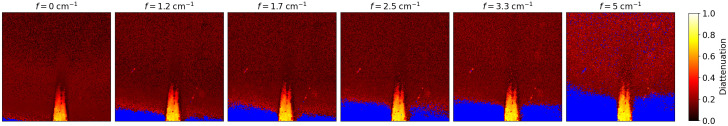
Diattenuation of the para-aramid fiber embedded in a PDMS-based scattering and absorbing phantom calculated using Lu-Chipman decomposition from the real part of the SFD Mueller matrix. Spatial frequency is listed above each image. Nonrealizable Mueller matrix pixels are depicted as blue. The spatial frequency sinusoidal pattern was oriented horizontally. The field of view of the images is 1.1  cm×1.1  cm.

The SFD Mueller matrix at $f=5\;{\mathrm{cm}}^{-1}$ in Fig. 2(b) is mostly, but not entirely, a physically realizable regular Mueller matrix (as determined by evaluating the eigenvalues of the covariance matrix). As pointed out earlier in Sec. 2, nonrealizability occurs when there is significant radiance emitted from one location when irradiance is applied at another. Because most of the image contains realizable matrices, we can apply Lu-Chipman decomposition to them and determine local diattenuation. Figure 3 shows the local diattenuation as a function of spatial frequency. The background regions shows some diattenuation, which we attribute to the off-axis geometry of the measurement, whereas the fibers show additional diattenuation, which we attribute to their alignment. Included in Fig. 3 is a blue mask showing those areas of the image which are not realizable. As one can see, the area of the sample for which the PDMS layer is thinnest yields nonrealizable behavior. We suspect that this occurs progressively in this region because the PDMS lies above a microscope slide, which separates the PDMS scattering medium from the diffusely scattering white tile under the slide. Note that the matrix is realizable in the region with the fibers. We believe that the fibers are more local scatterers, so that the signal from them is less dependent upon spatial frequency than that of the more diffusely scattering PDMS material.

The length of the fiber that is visible in the diattenuation images extends deeper into the PDMS as the spatial frequency is increased. Whereas SFDI is often used to reduce the probing depth, our observations suggests that the polarization information yields the opposite trend. Polarization preserving paths favor those which scatter the fewest times, and the combination of SFDI and MMP tends to enhance those paths.

### 6.2 Optical Fiber Bundle

**Fig. 4:**
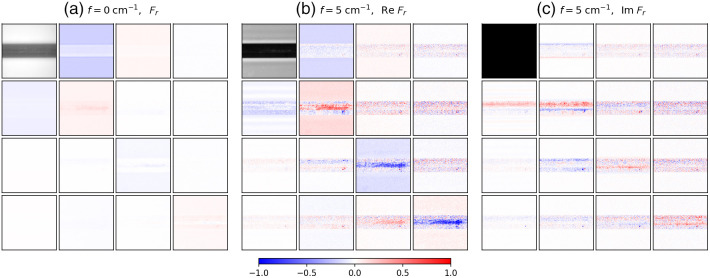
SFD Mueller matrix BRDFs from the optic fiber bundle: (a) the Mueller matrix BRDF at $f=0\;{\mathrm{cm}}^{-1}$, (b) the real part of the SFD Mueller matrix BRDF at $f=5\;{\mathrm{cm}}^{-1}$, and (c) the imaginary part of the SFD Mueller matrix BRDF at $f=5\;{\mathrm{cm}}^{-1}$. The maximum value of the black and white scale used for $F_{\text{r},11}$ at $f=0\;{\mathrm{cm}}^{-1}$ is six times the maximum value for $F_{\text{r},11}$ of $f=5\;{\mathrm{cm}}^{-1}$. The scale for the normalized SFD Mueller matrix elements is shown at the bottom of the figure. The spatial frequency sinusoidal pattern for $f=5\;{\mathrm{cm}}^{-1}$ was oriented horizontally. The field of view of the images is 1.1 cm × 1.1 cm.

**Fig. 5:**
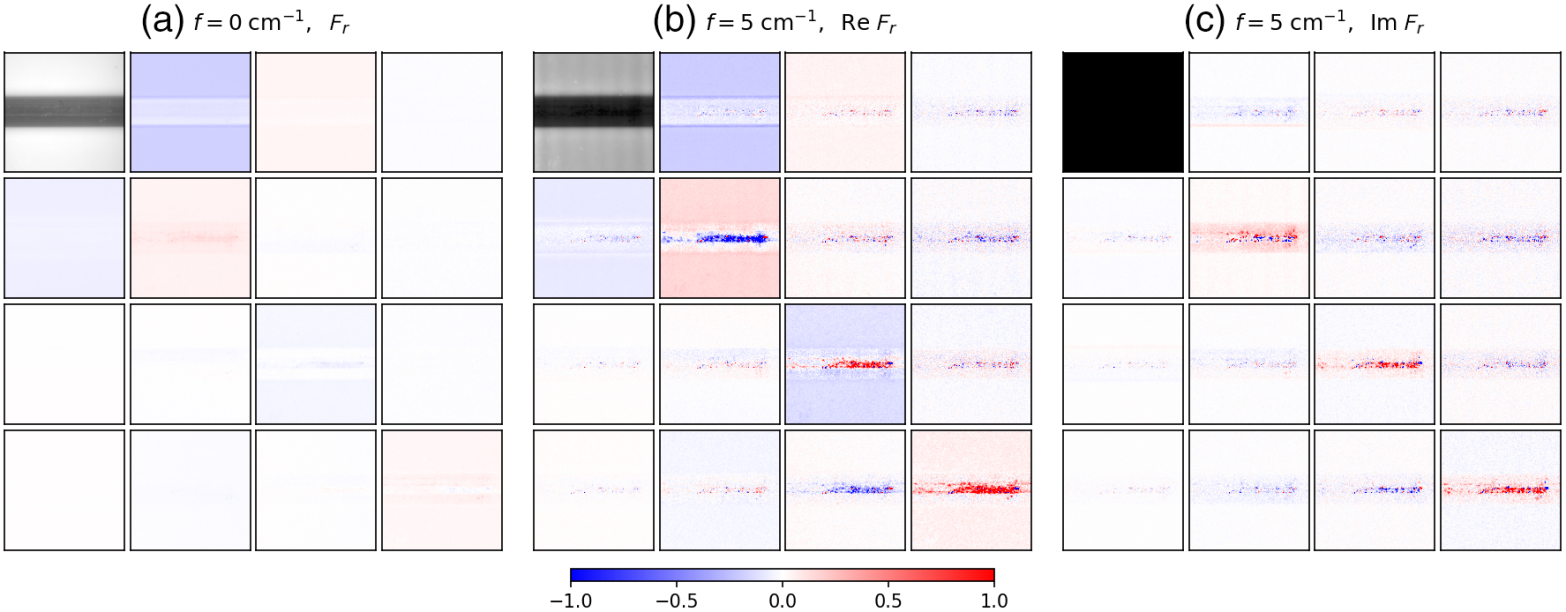
SFD Mueller matrix BRDFs from the optic fiber bundle: (a) the Mueller matrix BRDF at $f=0\;{\mathrm{cm}}^{-1}$, (b) the real part of the SFD Mueller matrix BRDF at $f=5\;{\mathrm{cm}}^{-1}$, and (c) the imaginary part of the SFD Mueller matrix BRDF at $f=5\;{\mathrm{cm}}^{-1}$. The maximum value of the black and white scale used for $F_{\text{r},11}$ at $f=0\;{\mathrm{cm}}^{-1}$ is seven times the maximum value for $F_{\text{r},11}$ of $f=5\;{\mathrm{cm}}^{-1}$. The scale for the normalized SFD Mueller matrix elements is shown at the bottom of the figure. The spatial frequency sinusoidal pattern for $f=5\;{\mathrm{cm}}^{-1}$ was oriented vertically. The field of view of the images is 1.1  cm×1.1  cm.

Figures 4 and 5 show the SFD Mueller matrix BRDF measured for the optical fiber bundle at $f=0\;{\mathrm{cm}}^{-1}$ and $f=5\;{\mathrm{cm}}^{-1}$, shown in the same manner as in Fig. 2, illuminated by the horizontal and vertical sinusoidal patterns, respectively. The image background is a glossy white ceramic. The results differ from those shown in Fig. 2 in that the SFD Mueller matrix has a significant imaginary component at the fiber bundle, especially when the spatial frequency pattern is oriented horizontally (see Fig. 4). The imaginary component arises, for example, because light incident on one side of the fiber bundle is emitted from the other side, both from scattering and from reflection inside the bundle. This behavior is less evident for the vertically oriented sinusoidal pattern (see Fig. 5), because this pattern selects light that is scattered and reflected horizontally, the opposite of the fiber bundle’s preferred scattering direction.

**Fig. 6:**
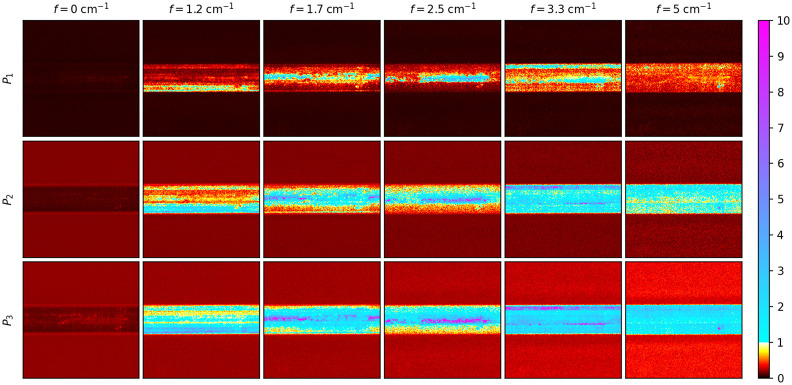
Polarimetric purity indices (rows) for each spatial frequency (columns) of an optic fiber bundle. The color scale is shown with a discontinuity at unity, differentiating those regions where the matrix is a valid regular Mueller matrix from those that are not. The spatial frequency sinusoidal patterns were oriented horizontally. The field of view of the images is 1.1  cm×1.1  cm.

**Fig. 7:**
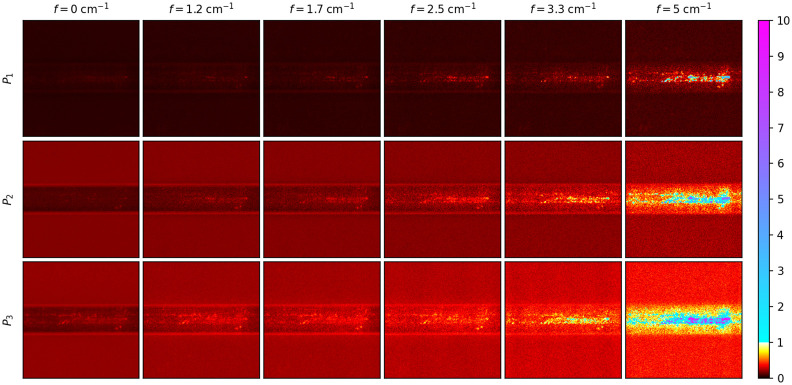
Polarimetric purity indices (rows) for each spatial frequency (columns) of an optic fiber bundle. The color scale is shown with a discontinuity at unity, differentiating those regions where the matrix is a valid regular Mueller matrix from those that are not. The spatial frequency sinusoidal patterns were oriented vertically. The field of view of the images is 1.1  cm×1.1  cm.

Figures 6 and 7 show the polarimetric purity indices ($P_{1},P_{2}$, and $P_{3}$, described in Sec. 2) calculated using only the real part of the matrices at the different spatial frequencies and the horizontally and vertically patterned illumination, respectively. The color scale for Figs. 6 and 7 was chosen to highlight those values that are less than one, compared with those greater than one. Values greater than one signify that the SFD Mueller matrix is not a valid regular Mueller matrix. Whereas the Mueller matrices at $f=0\;{\mathrm{cm}}^{-1}$ are entirely realizable, as expected, the matrices in the location of the fiber bundle are not realizable for even the smallest nonzero spatial frequency for horizontal sinusoidal illumination (see Fig. 6). Starting from $P_{1}$, values greater than one can be seen in the pixels that characterize the fiber bundle. The number of the nonrealizable pixels can be seen to increase with $P_{2}$ and $P_{3}$, where a majority of the fiber bundle is greater than one. The fraction of pixels within the fiber bundle having polarization purity indices greater than one also increases with spatial frequency for all three polarimetric purity indices. For vertical sinusoidal illumination, the imaginary parts are much weaker, and the fiber bundle remains mostly realizable until about $f=3.3\;{\mathrm{cm}}^{-1}$, where $P_{3}$ becomes greater than one along the fiber bundle’s core (see Fig. 7). $P_{3}$ of the fiber bundle is entirely greater than one by $f=5\;{\mathrm{cm}}^{-1}$. The background ceramic tile does not show nonrealizable behavior at the spatial frequencies shown. Due to the strong presence of nonrealizable Mueller matrices measured from the fiber bundle itself and uncertainty as to how such matrices should be interpreted, further Mueller matrix decomposition was not performed. These results show that SFD Mueller matrix imaging has contrast mechanisms that are very different than those observed in either SFDI or MMP alone.

### 6.3 Caprine Cerebellum

**Fig. 8:**
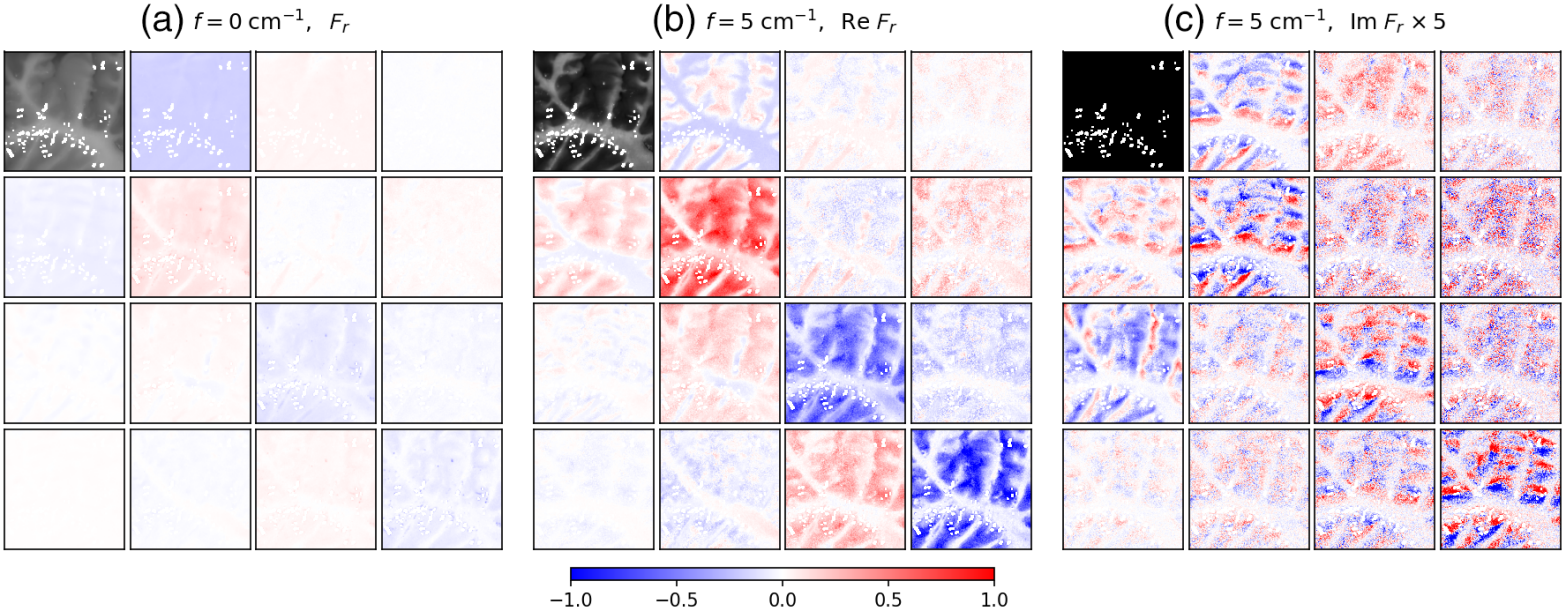
SFD Mueller matrix BRDF images of a caprine cerebellum: (a) the Mueller matrix BRDF at $f=0\;{\mathrm{cm}}^{-1}$, (b) the real part of the SFD Mueller matrix BRDF at $f=5\;{\mathrm{cm}}^{-1}$, and (c) the imaginary part of the SFD Mueller matrix BRDF multiplied by a factor of 5 at $f=5\;{\mathrm{cm}}^{-1}$. The maximum value of the black and white scale used for $F_{\text{r},11}$ at $f=0\;{\mathrm{cm}}^{-1}$ is four times the maximum value for $F_{\text{r},11}$ of $f=5\;{\mathrm{cm}}^{-1}$. The scale for the normalized SFD Mueller matrix elements is shown at the bottom of the figure. The spatial frequency sinusoidal pattern for $f=5\;{\mathrm{cm}}^{-1}$ was oriented horizontally. The field of view of the images is 1.1  cm×1.1  cm.

Figure 8 shows the SFD Mueller matrix BRDF images of the excised caprine cerebellum at $f=0\;{\mathrm{cm}}^{-1}$ and $f=5\;{\mathrm{cm}}^{-1}$, shown in the same manner as in Fig. 2. The imaginary part of the normalized SFD Mueller matrix elements were multiplied by a factor of 5 so that their features would be more visible on the same color scale used for the real part of the SFD Mueller matrices. Due to the moisture present, the unevenness, and curvature of the surfaces in the excised tissue, some areas of specular reflectance could not be avoided during the measurement. These localized and saturated pixels were masked and are seen as white pixels in the SFD Mueller matrix images. Specular reflection was less of an issue in our previous samples, where we were better able to mitigate it.

The branching structure observed in the images in Fig. 8 is white matter. Myelin sheaths that surround nerve fibers give those regions a different color and higher reflectance than the surrounding gray matter, which consists of cell bodies. The Mueller matrix at $f=0\;{\mathrm{cm}}^{-1}$ [see Fig. 8(a)] shows less contrast between the white and gray matters compared to the SFD Mueller matrix at $f=5\;{\mathrm{cm}}^{-1}$ [see Fig. 8(b)]. This difference in contrast is strongest between the $F_{\text{r},11}$ elements of both spatial frequencies where the delineation between the two tissue types improves with greater spatial frequency.^43^ The outline of the white matter can be seen to various degrees in the other Mueller matrix elements at $f=0\;{\mathrm{cm}}^{-1}$ but with less clarity compared to the higher SFD Mueller matrix elements. The gray matter in the real part of the SFD Mueller matrix at $f=5\;{\mathrm{cm}}^{-1}$ shows large magnitudes particularly along the matrix diagonal where the white matter maintains values close to zero.

Perhaps the most interesting feature for the cerebellum data is the imaginary component at $f=5\;{\mathrm{cm}}^{-1}$. Whereas the white matter exhibits only real SFD Mueller matrix values (imaginary part close to zero), the gray matter shows a striking pattern in contrast. The SFD Mueller matrix becomes complex-valued when the scattering is directionally unbalanced. The striped patterns seen in the imaginary part of the SFD Mueller matrix data appear to wrap around the borders of the white matter and are believed to be related to anisotropic scattering from the highly reflective white matter towards the gray matter. As stated previously the white matter is comprised of nerve fibers wrapped in myelin sheathes, making the white matter much more anisotropic in structure compared to the gray matter. The patterns observed in Fig. 8(c) show opposite signs above and below the white matter. For vertically oriented sinusoidal illumination, the pattern of opposite signs shows up left and right (not shown, but available in the online data).

### 6.4 Bovine Thalamus

**Fig. 9:**
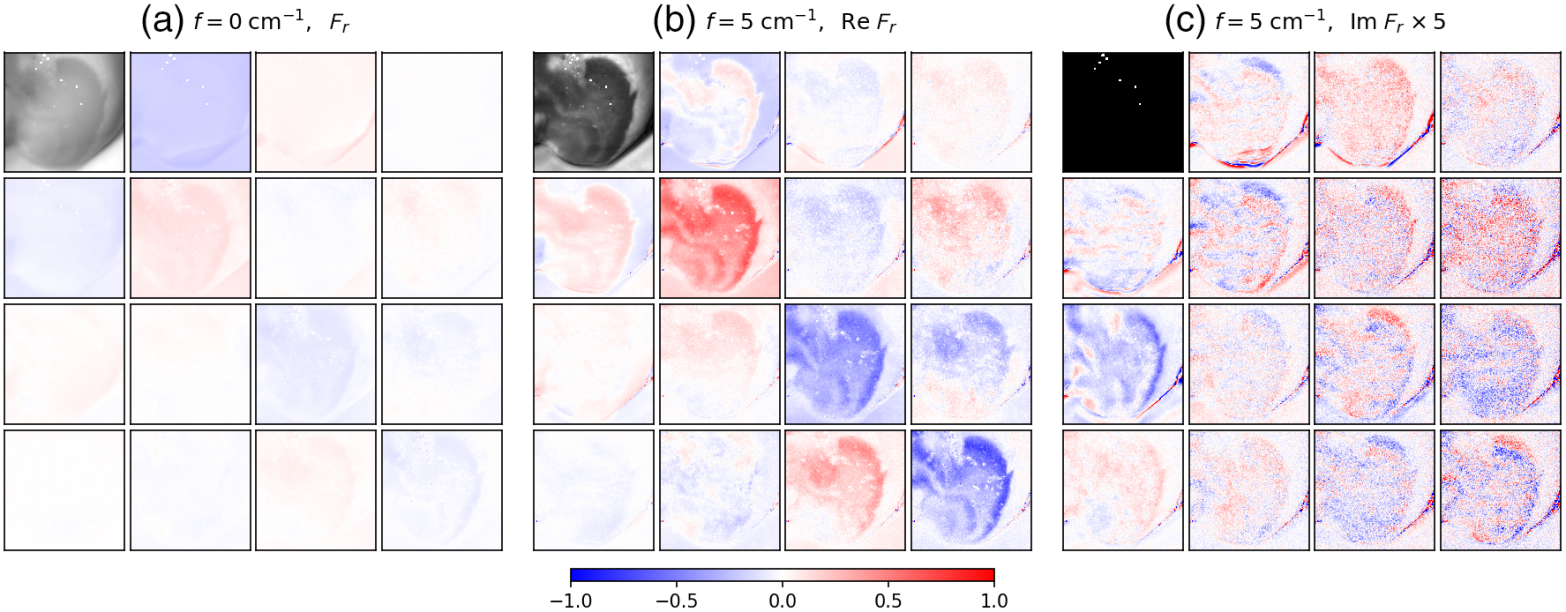
SFD Mueller matrix BRDF images of a bovine thalamus: (a) the Mueller matrix BRDF at $f=0\;{\mathrm{cm}}^{-1}$, (b) the real part of the SFD Mueller matrix BRDF at $f=5\;{\mathrm{cm}}^{-1}$, and (c) the imaginary part of the SFD Mueller matrix BRDF multiplied by a factor of 5 at $f=5\;{\mathrm{cm}}^{-1}$. The maximum value of the black and white scale used for $F_{\text{r},11}$ at $f=0\;{\mathrm{cm}}^{-1}$ is 3.5 times the maximum value for $F_{\text{r},11}$ of $f=5\;{\mathrm{cm}}^{-1}$. The scale for the normalized SFD Mueller matrix elements is shown at the bottom of the figure. The spatial frequency sinusoidal pattern for $f=5\;{\mathrm{cm}}^{-1}$ was oriented horizontally. The field of view of the images is 1.1  cm×1.1  cm.

Figure 9 shows SFD Mueller matrix BRDF images from the excised bovine thalamus at f=0 and $f=5\;{\mathrm{cm}}^{-1}$. The change in texture seen at the bottom of the images is the white background beneath the Petri dish that the brain tissue is resting on. Similar to the caprine cerebellum, both white and gray matter are present in the image area. Structurally, the thalamus is a mass of gray matter at the center of the mammalian brain with myelinated axons branching internally and externally from it to reach the surrounding brain areas. In Fig. 9, the contrast between the white and gray matter is significantly greater at $f=5\;{\mathrm{cm}}^{-1}$ compared with that at $f=0\;{\mathrm{cm}}^{-1}$. As with the cerebellum data, the white matter maintains a mostly real-valued SFD Mueller matrix (imaginary part negligible) at $f=5\;{\mathrm{cm}}^{-1}$. Interestingly, a marble pattern of white matter can be seen in the center of the gray matter of the $F_{\text{r},11}$ elements at $f=5\;{\mathrm{cm}}^{-1}$ that is difficult to observe at $f=0\;{\mathrm{cm}}^{-1}$. This white matter also maintains near-zero values in the SFD Mueller matrix element at $f=5\;{\mathrm{cm}}^{-1}$. This is likely part of the external medullary laminae, which are white matter structures that cover the lateral surface of the thalamus.^44^ Perhaps because the white matter is not so uniformly aligned in this sample, and because the spatial frequency is not high enough, there is not enough directional scattering to demonstrate as strong a pattern in the imaginary part of the SFD Mueller matrix compared with what was observed in the cerebellum data.

### 6.5 Bovine Cerebrum

**Fig. 10:**
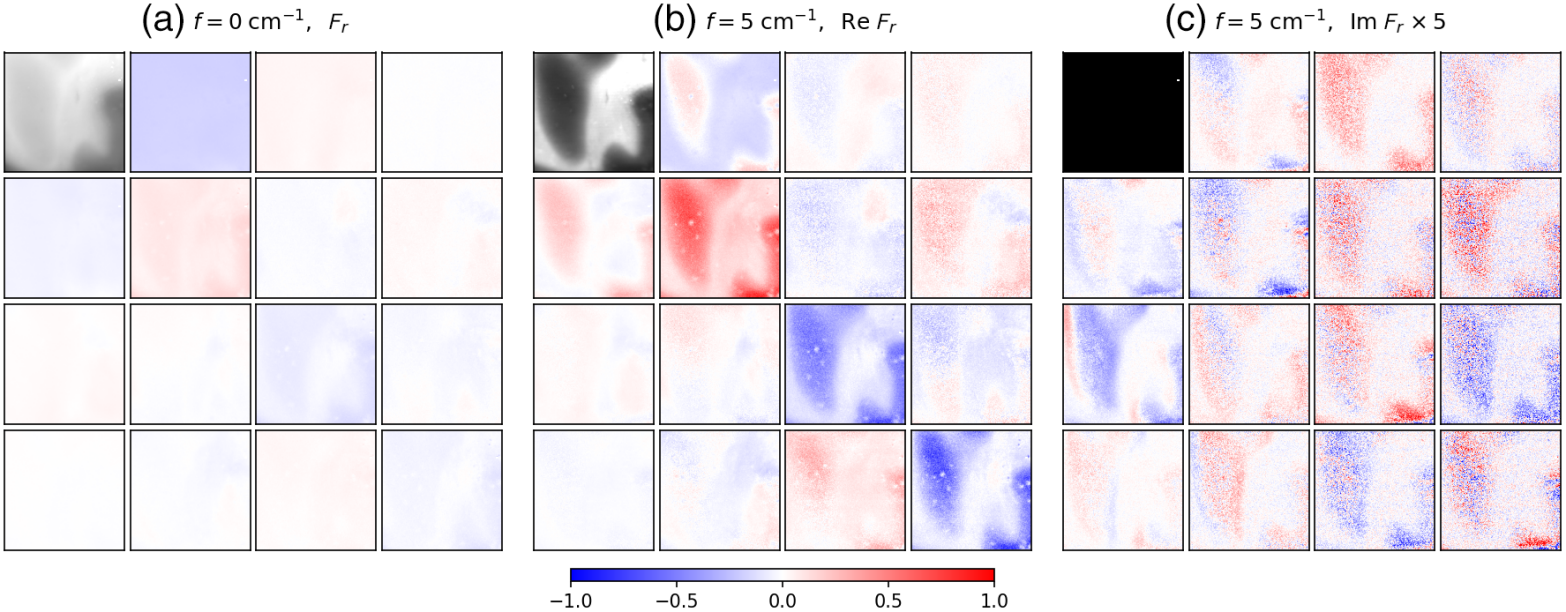
SFD Mueller matrix BRDF images of a bovine cerebrum: (a) the Mueller matrix BRDF at $f=0\;{\mathrm{cm}}^{-1}$, (b) the real part of the SFD Mueller matrix BRDF at $f=5\;{\mathrm{cm}}^{-1}$, and (c) the imaginary part of the SFD Mueller matrix BRDF multiplied by a factor of 5 at $f=5\;{\mathrm{cm}}^{-1}$. The maximum value of the black and white scale used for $F_{\text{r},11}$ at $f=0\;{\mathrm{cm}}^{-1}$ is three times the maximum value for $F_{\text{r},11}$ of $f=5\;{\mathrm{cm}}^{-1}$. The scale for the normalized SFD Mueller matrix elements is shown at the bottom of the figure. The spatial frequency sinusoidal pattern for $f=5\;{\mathrm{cm}}^{-1}$ was oriented horizontally. The field of view of the images is 1.1  cm×1.1  cm.

Figure 10 shows SFD Mueller matrix images from the inner surface area of the excised bovine cerebrum at $f=0\;{\mathrm{cm}}^{-1}$ and $f=5\;{\mathrm{cm}}^{-1}$. Like the caprine cerebellum and the bovine thalamus, white matter and gray matter are distinguishable. At $f=0\;{\mathrm{cm}}^{-1}$, the $F_{\text{r},11}$ image is dominated by white matter, except for the lower right section of the image. The SFD Mueller matrix at $f=5\;{\mathrm{cm}}^{-1}$ shows areas of gray matter beneath the less dense sections of white matter that were not visible at $f=0\;{\mathrm{cm}}^{-1}$. The borders between the white and gray matter are stronger for this SFD Mueller matrix compared with the $f=0{\;\mathrm{cm}}^{-1}$ Mueller matrix. This delineation is most apparent in the diagonal of the real part of the SFD Mueller matrix at $f=5\;{\mathrm{cm}}^{-1}$, where the white matter retains a value close to zero and the gray matter makes up most of the imaginary component.

## 7 Discussion

The SFD Mueller matrix images shown in Sec. 6 demonstrate contrast mechanisms that differ from those observed in either SFDI or regular Mueller matrix imaging. In most of our results, the SFD Mueller matrix has higher contrast as the spatial frequency is increased. This finding highlights how combining MMP with SFDI can facilitate measurements of the polarimetric properties of the surfaces of diffusively scattering materials, which is difficult to measure with MMP alone.

There are other aspects of the results which we can point out and qualitatively understand. Scattering can be treated generally as the sum of contributions from single scatter, double scatter, and diffusive scatter (those paths that scatter three or more times). Single scatter should exhibit a flat SFD response, because the point spread function associated with it is highly localized. In addition, single scatter should yield a real-valued SFD Mueller matrix that obeys the realizability rules for regular Mueller matrices. We observe this in the PDMS-based scattering phantom in the region where the para-aramid fibers are only shallowly embedded (see Figs. 2 and 3).

Diffusive scatter is expected to be highly depolarizing. Some polarizing behavior can arise from the polarization dependence of Fresnel transmission in and out of the material and the polarization dependence of the first and last scattering events.^45^ We can see this in the data, especially for the background materials (PDMS in Fig. 2 or ceramic in Figs. 4 and 5) in many of the images. Because the incident radiation is incident at a 20 deg angle and viewed from the surface normal, we observe diattenuation (as evident by negative values for $M_{12}$) consistent with this geometry, where p-polarization has higher transmittance into the material.

Double scatter involves the spatial separation between two scattering events. For horizontal sinusoidal irradiance, if the two scatter events are vertically displaced, there would be a phase imparted in the SFD image if there is more scattering upwards than downwards or vice versa. In unpolarized SFD imaging, it is impossible to separate phase shifts that occur due to topography from those that occur due to the directionality of the scatter. However, with SFD Mueller matrix imaging, when one polarization scatters more than another in a geometrically unbalanced fashion, these phase shifts can be distinguished. For example, consider the fiber bundle data shown in Fig. 5(c), where there is a strong imaginary component to the SFD Mueller matrix. From Fig. 6, the SFD Mueller matrix (as reduced to polarization purity indices) varies significantly with spatial frequency. This strong spatial frequency dependence results from the matching of the irradiance period with the dimensions of the fiber bundle and the multiple internal reflections in it. This effect does not occur as strongly when the sinusoidal irradiation is perpendicular to the fiber bundle (compare Figs. 6 with 7). Thus, SFD Mueller matrix imaging allows the directional scattering within a medium to be probed.

These directional effects of the scattering can also be observed in the imaginary part of the SFD Mueller matrix measured from the various brain specimens, especially the caprine cerebellum shown in Fig. 8(c). Whereas the imaginary part of the SFD Mueller matrix is weak in the regions containing white matter, it tends to have significant values in neighboring gray matter. For example, in Im Fr,22, in areas immediately above the white matter, the values are negative, while in those below, the values are positive. The change in the sign above and below the white matter results from the displacement of the photon transfer being in opposite directions, with the polarization behavior otherwise being the same. These effects can be observed to a lesser extent in the bovine thalamus data in Fig. 9. However, because the length scales for the white matter features in the thalamus are smaller and the length associated with the spatial frequency is long, the effect is relatively weak.

At this time, the analysis is hampered by the lack of a framework for characterizing the resulting matrices. As mentioned in Sec. 2, the properties of a SFD Mueller matrix are not the same as a regular Mueller matrix, in that it does not express the relationship between a Stokes vector irradiance and a Stokes vector radiance. Because a SFD Stokes vector radiance does not follow Eq. (2) and because it is complex-valued, the SFD Mueller matrix does not have the same requirements as a regular Mueller matrix. Thus, it is not straightforward to apply decomposition methods, such as Eq. (18), to the results. It is expected that diattenuation angles and retardance angles could be extracted from data using these traditional methods, because rotation transformations are not expected to change in the SFD. However, the current understanding of the mathematics of depolarization is intimately related to the requirement that the eigenvalues of the covariance matrix H be positive. Thus, further work needs to go into developing the analysis infrastructure to fully interpret the results.

## 8 Summary

We demonstrated an imaging modality that combines MMP and SFDI. We demonstrated theoretically that the combined SFD Mueller matrix imaging at nonzero frequency is best described as a complex-valued matrix, having phase and amplitude information and that this phase information can be differentiated from that caused by topography or shape. Whereas this measurement method is very much in its infancy, we show that it has potential for distinguishing the polarization behavior for different photon path lengths and scattering directions in a material. This imaging modality may have applications in the field of biomedical imaging where thick and structurally complex tissues are commonplace. Depending on the tissue type, selecting for photon path length can potentially highlight different structures such as how the contrast between the white matter and gray matter improved with increased spatial frequency in the brain samples shown. Brain tissue has been the subject of recent interest in the field of MMP.^8^ Mueller matrix imaging has been used to observe changes that occur during pregnancy or disease in cervical collagen anisotropy.^46^ One difficulty in imaging cervical collagen is that it is surrounded by other tissues of lesser interest. Imaging modalities that allow for selection criteria for what is imaged may have potential in similar settings. Complicating the analysis and interpretation is the finding that the SFD Mueller matrix is inherently complex-valued and does not represent a realizable regular Mueller matrix. However, it is observed that contrast to different features in the image can be enhanced and that single and multiply scattered paths can be distinguished. Future work will continue to explore the utility of this imaging method for biomedical applications.

## Data Availability

The data presented in this manuscript are available for download online at the NIST Public Data Repository, https://doi.org/10.18434/mds2-2694.
